# Correlation between L-Lactate Concentrations in Beef Cattle, Obtained Using a Hand-Held Lactate Analyzer and a Lactate Assay Colorimetric Kit

**DOI:** 10.3390/ani11040926

**Published:** 2021-03-25

**Authors:** Daniela M. Meléndez, Sonia Marti, Luigi Faucitano, Derek B. Haley, Timothy D. Schwinghamer, Karen S. Schwartzkopf-Genswein

**Affiliations:** 1 Agriculture and Agri-Food Canada, Lethbridge Research and Development Centre, Lethbridge, AB T1J 4B1, Canada; daniela.melendezsuarez@canada.ca (D.M.M.); timothy.schwinghamer@canada.ca (T.D.S.); 2 IRTA Ruminant Production Program, Caldes de Montbui, 08140 Barcelona, Spain; sonia.marti@irta.cat; 3 Agriculture and Agri-Food Canada, Sherbrooke Research and Development Centre, Sherbrooke, QC J1M 0C8, Canada; luigi.faucitano@canada.ca; 4 Department of Population Medicine, University of Guelph, Guelph, ON N1G 2W1, Canada; dhaley@uoguelph.ca

**Keywords:** analysis, blood, cattle, L-lactate, transport

## Abstract

**Simple Summary:**

Lactate is a metabolite used in animal research as an indicator of muscle fatigue; therefore, it has been used as an indicator of cattle response to long distance transportation. The aim of this study was to assess the relationship of L-lactate concentrations measured using a Lactate Scout+ hand-held analyzer and a traditional lactate assay colorimetric kit. Blood samples were collected from 96 steers prior to loading and after 36 h of transportation, and prior to reloading and after an additional 4 h of road transportation, and on d 1, 2, 3, 5, 14, and 28 after transport. The Lactate Scout+ hand-held analyzer strip was dipped in blood at the time of sampling, while blood samples were collected into sodium fluoride tubes for use in the colorimetric analysis. Correlations were calculated to assess the strength of the relationship between the L-lactate concentrations measured between methods. The strength of the correlation and the level of statistical significance varied over the observed time points, while the correlation for the pooled data was weak. Based on the low strength of the correlation, the Lactate Scout+ analyzer is not a suitable alternative to a colorimetric assay for measuring L-lactate in transported cattle.

**Abstract:**

Lactate is a product of anaerobic glycolysis, used in animal research as an indicator of muscle fatigue. Therefore, it has been used as an indicator of cattle response to long distance transportation. The aim of this study was to assess the relationship of L-lactate concentrations measured using a Lactate Scout+ analyzer and a traditional lactate assay colorimetric kit. Blood samples were collected by venipuncture from 96 steers (Black or Red Angus × Hereford/Simmental and Black or Red Angus × Charolais; 247 ± 38.2 kg BW) prior to loading (LO1) and after 36 h of transport, and prior to reloading and after an additional 4 h of road transportation, and on d 1, 2, 3, 5, 14, and 28 after transport. The Lactate Scout+ analyzer strip was dipped in blood at the time of sampling, while blood samples were collected into sodium fluoride tubes for use in the colorimetric analysis. Pearson correlations were calculated to assess the strength of the relationship between the experimental methods for the quantification of L-lactate concentrations. The magnitude and direction of the correlation, and the level of statistical significance varied over the observed time points, ranging from *r* = −0.03 (*p* = 0.75; LO1) to *r* = 0.75 (*p* < 0.0001; d 3). The correlation for the pooled data was weak but statistically significant (*r* = 0.33, *p* < 0.0001). Based on the low magnitude of the correlation due to variability across sampling time points in this study, the Lactate Scout+ analyzer is not a suitable alternative to a lab-based assay (considered the gold standard) for measuring L-lactate in transported cattle.

## 1. Introduction

Lactate is a metabolite that exists as two stereoisomers, namely the L (+) lactate, produced by mammalian cells, and the D (−) lactate, produced by bacteria in the gastrointestinal tract [1]. Under anaerobic conditions, L-lactate is produced in all tissues as an end product of glycolysis, but is produced more abundantly in the renal medulla, skeletal muscle, red blood cells, and brain [2]. 

In clinical settings, L-lactate was measured as a factor of prognosis and diagnosis of a variety of human and animal health conditions such as shock, severe sepsis, low cardiac output, liver failure, neoplasia, poisoning, and drug therapy [1,3,4]. In animal science studies, the analysis of blood lactate concentrations have been used to assess the effects of preslaughter handling (pigs [5,6], cattle [7,8], sheep [9], and trout: [10]) and transport stress (cattle: [11], poultry: [12], and pigs [13]). 

L-lactate can be measured using methodologies, such as commercially available enzymatic analytical assays, blood gas analyzers, or hand-held devices, with the latter ones being developed to be more time and cost effective than traditional analytical methods. Lactate Scout hand-held monitors have been validated for use in horses [14], dogs [15], sheep [16], pigs [17], cows, and calves [18]. Hand-held analyzers, such as the Accusport (Boheringer, Mannheim, Germany), Accutrend Plus (Roche Diagnostics, Mannheim, Germany), i-STAT (Abbott Point of Care, Abbott Laboratories, Chicago, IL), Lactate Scout (SensLab GmbH, Leipzig, Germany), and Lactate Pro (Arkay Inc, Kyoto, Japan) were shown to correlate (*r* > 0.94) with the laboratory based measures in cattle with different diseases [19,20]. To our knowledge, the Lactate Scout+ hand-held analyzer (SensLab GmbH, Leipzig, Germany) has not been validated for assessing muscle fatigue in response to transport stress and handling in cattle.

Therefore, the aim of this study was to assess the correlation between L-lactate concentrations measured using a Lactate Scout+ analyzer and a lactate assay colorimetric kit in cattle exposed to varying pre-transport management as well as varying rest stop durations.

## 2. Materials and Methods

This protocol was approved by the Animal Care Committee of the Lethbridge Research and Development Centre (LeRDC) (ACC number 1918). Animals were cared for in accordance with the Canadian Council of Animal Care [21]. 

Animals sampled in the present study were part of a larger study focused on assessing the effect of conditioning, source and rest during transport on indicators of welfare in beef cattle [22]. As described in the previous study, Ninety-six (96) crossbred (Black or Red Angus × Hereford/Simmental and Black or Red Angus × Charolais) steer calves 247 kg ± 38.2 kg of BW were sourced from two different ranches in southern Alberta (Canada). Treatments were distributed according to a 2 × 2 × 2 factorial design, where the main factors included conditioning: conditioned (C; *n* = 12) or non-conditioned (NC; *n* = 12) calves; source: auction market (A; *n* = 12) or ranch direct (R; *n* = 12) calves; and rest stop: 0 h (*n* = 12) or 8 h (*n* = 12) of rest. Calves were randomly assigned to treatments and pens. The NC calves received an *ad libitum* diet of 35% corn silage, 20% alfalfa hay, 12% barley grain and 3% supplement with vitamins and minerals the first three days after arrival to the feedlot, while the C calves received an 85% corn silage, 12% barley grain and 3% supplement with vitamins and minerals diet. After the three day adaptation period the NC calves received the same diet as the C calves. Calves in the present study were castrated between 2 to 3 months of age at the ranch of origin, and calves were transported after weaning when calves were 7 to 8 months of age. 

Calves were transported by road in cattle tri-axle trailer liners with bedding and no access to feed and water during transport. Calves were transported on days in which ambient temperatures ranged between −11.9 and 19.8 °C. Long distance (>24 h) transportation is well known to stimulate lactate production and therefore transportation was used as the model to measure and compare differences in L-lactate production. Calves were blood sampled prior to loading (LO1) and after unloading (UN1) following a 36 h transport, and prior to loading (LO2) and after unloading (UN2) following an extra 4 h transport. In addition, calves were sampled at d 1, 2, 3, 5, 14, and 28 after UN2 to assess the recovery rate after long transportation. The 0 h calves were unloaded and only sampled at UN1 after 36 h of transport, however these animals did not have a loading (LO2) sampling point prior to the additional 4 h transport because they were not rested. 

Blood samples were collected through jugular venipuncture into a 7-mL sodium fluoride tube without anticoagulant (BD vacutainer; Becton Dickinson Co., Franklin Lakes, NJ) and left at room temperature for at least 1 to 2 h prior to centrifugation for 15 min at 2.5× *g* at 4 °C. Serum was decanted and frozen at −80 °C for further analysis. The method used as the gold standard in the current study was the colorimetric assay kit. Lactate was measured using a L-Lactate colorimetric assay (Lactate Assay Kit, Cell Biolabs, Inc., San Diego, CA) in which lactate oxidase converted lactate into pyruvate and hydrogen peroxide. The hydrogen peroxide was then detected with a colorimetric probe. The intra- and inter-assay coefficients of variation (CV) for the blood lactate concentration assay were 3.7 and 1.9%, respectively. The standard curve of all assays had an *R*^2^ of 0.99. Hemolytic samples were not analyzed. 

This study was not a standalone study and was done as part of a large study assessing physiological and behavioural indicators of stress associated with transport and rest periods in calves. At the time of sampling, a strip (one per sample) was inserted into the Lactate Scout+ hand-held device and dipped in a fresh blood sample. Based on study logistics it was not possible to collect duplicate samples at the time. The concentrations of L-lactate were determined through an enzymatic amperometric system using lactate oxidase. The Lactate Scout+ values ranged from 0.5 to 25 mmol/L. 

### Statistical Analysis

All statistical procedures performed in the current study were carried out using the Statistical Analysis Software (SAS, version 9.4, SAS Inst. Inc., Cary, NC, USA). Pearson’s product-moment correlations were calculated to quantify the strength and direction of the relationship between L-lactate concentrations obtained via the Lactate Scout+ versus those obtained via the colorimetric assay for each sampling point. Correlations were calculated for blood lactate values resulting from different treatments and obtained at all sampling points. A paired *t*-test was used to determine the mean difference between the two methods for each animal. 

Samples (888 samples) were divided evenly (*n* = 296) into low (0.17 to 0.61 mmol/L), medium (0.62 to 1.05 mmol/L) and high (1.06 to 2.20 mmol/L) lactate concentrations using the colorimetric test values (0.17 to 2.20 mmol/L). Correlations were calculated for low, medium, and high concentrations separately to assess if correlations differed between concentration groups.

In addition, lactate concentrations obtained using the Lactate Scout+ and the colorimetric kit were analyzed separately as a repeated measures factorial design [22]. Data were analyzed using mixed models due to the inclusion of fixed effects: conditioning, source, and time (nested in rest) and random effects: animal and pen. Time was nested in rest to account for the missing sampling point (LO2) for the 0 h treatment calves, which did not receive a rest. To account for breed differences, breed was added as a covariate to the statistical model. Results are reported as least squares-means (*µ*) including the upper (u) and lower (l) limits at a 95% confidence. Data were analyzed using PROC GLIMMIX (SAS, version 9.4, SAS Inst. Inc., Cary, NC, Canada). Statistical significance was *p* ≤ 0.05.

## 3. Results

The strengths and levels of statistical significance of the correlation between methods varied across sampling time points (Table 1) and treatments (Table 2). The correlation for the pooled data was weak but statistically significant (*r* = 0.33, *p* < 0.001). To account for the lactate concentrations not being collected in duplicate, a comparison was done between the Lactate Scout+ values and the values obtained from either one of the wells of the laboratory kit. The correlation between the Lactate Scout+ values and the colorimetric kit average (*r* = 0.33, *p* < 0.001) was similar to the correlation between the Lactate Scout+ values and the values of well 1 (*r* = 0.33, *p* < 0.001) and well 2 (*r* = 0.32, *p* < 0.001).

**Figure 1:**
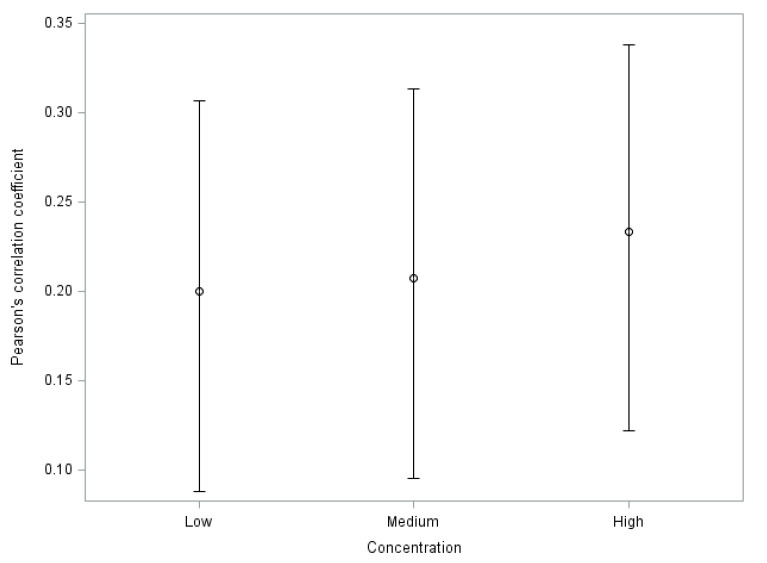
Values of Pearson’s product-moment correlation coefficient (*r*). Whiskers represent intervals at 95% confidence for low, medium, and high lactate concentration groups.

Correlations observed for low (*r* = 0.20, *p* < 0.001), medium (*r* = 0.20, *p* < 0.001), and high (*r* = 0.22, *p* < 0.001) concentrations were similar among the three groups, where a weak but statistically significant correlation was observed (Figure 1). These results show a robust correlation for the three groups. 

The paired *t*-test showed that L-lactate concentrations from the Lactate Scout+ and the colorimetric assay were significantly different (*p* < 0.0001). L-lactate concentrations measured with the Lactate Scout+ ranged between 0.3 and 15.3 mmol/L (mean = 1.7 mmol/L, standard deviation = 1.37 mmol/L) while the colorimetric assay concentrations ranged between 0.17 and 2.20 mmol/L (mean = 0.91 mmol/L, standard deviation = 0.45 mmol/L). The concentration of L-lactate was overestimated (range between 0.1 and 13.6 mmol/L) by Lactate Scout+ for the majority of the samples (791 out of 888) compared to the colorimetric assay. Differences in L-lactate concentrations between methods ranged between −1.20 and 13.63 mmol/L with a median difference of 0.46 mmol/L. 

The results of the factorial design did not follow the same trends for lactate concentrations measured using the Lactate Scout+ or the colorimetric kit. Lactate concentrations using the Lactate Scout+ showed a conditioning effect (*p* < 0.01), where C calves (*µ* = 1.7 mmol/L (u = 2.11, l = 1.45)) had greater (*p* < 0.01) lactate concentrations than N calves (*µ* = 1.4 mmol/L (u = 1.70, l = 1.18)). No differences (*p* < 0.01) were observed for source, rest (nested in time) or their interactions. Contrary to the previous results, a source × conditioning × rest (nested in time) effect (*p* < 0.01) was observed for the lactate concentrations measured using the colorimetric kit. The C-R−8h calves had greater lactate concentrations than the N-R-8h calves at UN1 and d 14 (C-R-8h: UN1 *µ* = 1.7 mmol/L (u = 1.91, l = 1.40), d14 *µ* = 1.0 mmol/L (u = 1.23, l = 0.71) and N-R-8h: UN1 *µ* = 1.1 mmol/L (u = 1.35, l = 0.85), d 14 *µ* = 0.6 mmol/L (u = 0.86, l = 0.35)). 

## 4. Discussion

The comparison with the colorimetric assay reference method showed that lactate concentrations obtained with the Lactate Scout+ varied across treatments and sampling time points. According to the Lactate Scout+ manual, the acceptable hematocrit range is 20–70%. In the present study, the observed hematocrit values of calves ranged between 18% and 43%, which is slightly below the acceptable Lactate Scout+ hematocrit range. Nevertheless, this does not explain the variability in the results because only 3 out of 888 samples had a hematocrit lower than 20% and one of the features of the Lactate Scout+ is that it compensates for hematocrit out of the recommended range. Therefore, hematocrit cannot explain the low correlation observed between methods. 

According to the Lactate Scout+ manual, the analyzer works between 5 and 45 °C and 10 and 85% humidity. During the trial, temperature and humidity outside the barn ranged between −11.4 and 16.5 °C and 37 and 95%, respectively. Although sampling was done inside a heated barn, which was within the manufacturers suggested temperature range, occasionally wind entering the barn may have decreased the temperature below 5 °C, potentially affecting the accuracy of the Lactate Scout+ hand-held analyzer. Therefore, data collected on days when the temperature and humidity (outside the barn) were above or below the recommended range (438 data points out of a total of 888) were removed from the data set and the data was reanalyzed. The correlation from the subset of data points was similar to that obtained for the entire data set (*r* = 0.37 vs. *r* = 0.33; *p* < 0.001 for both). Based on these results, temperature and humidity do not explain the difference observed between the two blood lactate analytical methods. 

Results of the present study did not confirm findings from previous studies validating the Lactate Scout. In a previous study, where methods of blood plasma sample storage pending lab analysis using traditional enzymatic kits (Trinity Biotech, Bray, Ireland) were assessed, a strong correlation (*r* = 0.98) was reported between blood lactate concentration obtained using an enzymatic method and the Lactate Scout hand-held analyzer after samples were stored at 4 and 20 °C and analyzed 0, 30, 60, 120, and 240 min after collection [15]. Whereas, in other studies, strong correlations (*r* > 0.97) were reported between blood lactate concentrations obtained using hand-held analyzers and laboratory methods for plasma samples stored at −20 °C (dogs [18] and cattle [20]). In the present study, samples were kept at room temperature for at least 1 to 2 h, were centrifuged, and serum was decanted and stored at −80 °C for a period of 66 to 85 days before analysis. Although storage temperature differs between our study and the previously mentioned studies, serum metabolites have been reported to be more stable when stored at −70 than −20 °C [23,24]. Based on the previously mentioned studies, handling prior to centrifugation and storage temperature after centrifugation do not explain the differences observed between methods.

With the exception of one study [14], animals in all previous validation studies were sampled once, while animals in this study were sampled a total of 10 times. Castagnetti et al. [14] sampled horses a similar number of times (seven) to those in the present study and reported a strong correlation (*r* = 0.95; *p* < 0.01) between plasma lactate concentrations obtained using the Lactate Scout and an enzymatic colorimetric assay. Therefore, sampling frequency does not help to explain the low correlations between methods or the variation observed across treatments and sampling time points observed in the present study. 

To our knowledge, there are no studies validating the Lactate Scout+ analyzer in animals or humans. Results of the present study did not confirm findings from previous studies validating the Lactate Scout (previous model to the Lactate Scout+). The device used in the earlier validation studies was an older model (Lactate Scout, SensLab GmbH, Leipzig, Germany) that was validated in pigs [17], horses [14], dogs [25], sheep [16], and cattle [18,20]. The Lactate Scout+ is a newer model than the Lactate Scout, and its novel features include a compensatory mechanism for differences in hematocrit and integrated Bluetooth technology. These results suggest that changes made to the new model (Lactate Scout+) may have resulted in the reduced correlation of lactate concentrations obtained with the Lactate Scout+ compared to the laboratory assay method. Differences in erythrocytes between humans and other species as well as the proprietary algorithms used to calculate lactate concentrations have been suggested as potential factors that could affect the accuracy of hand-held lactate analyzers [26]. We do not know if this is the reason why the Lactate Scout+ model has been discontinued by the manufacturer at this time. The newest model of hand-held analyzer, the Lactate Scout 4 (Lactate Scout 4, SensLab GmbH, Leipzig, Germany), has both the hematocrit compensatory mechanisms and the integrated Bluetooth technology. 

A limitation from the present study was that L-lactate concentrations were not collected in duplicate when utilizing the Lactate Scout+; therefore, it was not possible to assess the reliability of the hand-held analyzer. 

## 5. Conclusions

The results of the present study suggest that the concentrations obtained with the hand-held Lactate Scout+ are not comparable to concentrations obtained with the laboratory method, possibly due to a lack of replicates when measuring lactate using the Lactate Scout+, or due to technical problems with the new Lactate Scout+ model. New technology hand-held analyzers need to be validated prior to use in research or clinical settings. 

## Figures and Tables

**Table 1:** Pearsons’s product-moment correlation between Lactate Scout+ analyzer and a colorimetric assay at each sampling point (pooled data).

Sampling Point ^1^	*r*	*p*-Value
LO1	−0.03	0.74
UN1	0.37	<0.01
LO2	0.61	<0.01
UN2	0.59	<0.01
d1	0.37	<0.01
d2	0.64	<0.01
d3	0.75	<0.01
d5	0.28	<0.01
d14	0.69	<0.01
d28	0.64	<0.01

^1^ LO1: before loading; UN1 at unloading after 36 h transport; LO2: before the second loading; UN2: at the second unloading after the additional 4 h transport; d: day after transport.

**Table 2:** Pearsons’s product moment correlation between Lactate Scout+ and a colorimetric assay for each treatment at each sampling point.

	Treatment ^1^							
Sampling Point ^2^	C-R-0h	C-R-8h	C-A-0h	C-A-8h	NC-R-0h	NC-R-8h	NC-A-0h	NC-A-8h
LO1	0.37	−0.02	0.56	0.02	−0.11	−0.52	0.24	0.43
	0.23	0.94	0.05	0.93	0.71	0.07	0.43	0.15
UN1	0.75	−0.05	0.28	0.41	0.49	0.78	0.59	0.55
	<0.01	0.86	0.37	0.18	0.10	<0.01	0.04	0.06
LO2	*	0.39	*	0.59	*	0.26	*	0.81
		0.19		0.03		0.40		<0.01
UN2	0.49	0.54	0.40	0.41	0.81	0.69	0.68	0.76
	0.09	0.06	0.18	0.18	<0.01	0.01	0.01	<0.01
d1	0.45	0.82	0.87	0.50	0.60	0.76	0.51	0.73
	0.13	<0.01	<0.01	0.09	0.03	<0.01	0.08	<0.01
d2	0.67	0.80	0.49	0.23	0.78	0.86	0.72	0.85
	0.01	<0.01	0.09	0.46	<0.01	<0.01	<0.01	<0.01
d3	0.84	0.83	0.78	0.66	0.10	−0.10	0.82	0.98
	<0.01	0.03	<0.01	0.15	0.74	0.84	<0.01	<0.01
d5	0.49	0.92	−0.03	0.04	0.71	0.74	0.24	−0.27
	0.10	<0.01	0.91	0.90	<0.01	<0.01	0.43	0.39
d14	0.69	0.66	0.49	0.69	0.29	0.33	0.77	0.96
	0.01	0.01	0.10	0.01	0.36	0.29	<0.01	<0.01
d28	0.85	0.74	0.78	0.65	0.80	0.48	0.75	0.77
	<0.01	<0.01	<0.01	0.02	<0.01	0.11	<0.01	<0.01

* Missing data due to lack of UN2 sampling point for 0 h. ^1^ C: Conditioned calves, NC: Non-conditioned calves, R: ranch direct calves, A: auction market calves, 0 h: no rest, and 8 h of rest. ^2^ LO1: before loading; UN1 at unloading after 36 h transport; LO2: before the second loading; UN2: at the second unloading after the additional 4 h transport; d: day after transport.

## Data Availability

The data presented in this study is available upon request from the corresponding author.

## References

[1] Pang D.S., Boysen S. (2007). Lactate in veterinary critical care: Pathophysiology and management. J. Am. Anim. Hosp. Assoc..

[2] Fall P.J., Szerlip H.M. (2005). Lactic acidosis: From sour milk to septic shock. J. Intensive Care Med..

[3] Chrusch C., Bands C., Bose D., Li X., Jacobs H., Duke K., Bautista E., Eschun G., Bruce Light R., Mink S.N. (2000). Impaired hepatic extraction and increased splanchnic production contribute to lactic acidosis in canine sepsis. Am. J. Respir. Crit. Care Med..

[4] Revelly J., Tappy L., Martinez A., Bollmann M., Cayeux M., Berger M.M., Chioléro R.L. (2005). Lactate and glucose metabolism in severe sepsis and cardiogenic shock. Crit. Care Med..

[5] Warriss P.D., Brown S.N. (1994). A survey of mortality in slaughter pigs during transport and lairage. Vet. Rec..

[6] Brandt P., Aaslyng M.D. (2015). Welfare measurements of finishing pigs on the day of slaughter: A review. Meat Sci..

[7] Warner R., Ferguson D., Cottrell J., Knee B. (2007). Acute stress induced by the preslaughter use of electric prodders causes tougher beef meat. Aust. J. Exp. Agric..

[8] Gruber S., Tatum J., Engle T., Chapman P., Belk K., Smith G. (2010). Relationships of behavioral and physiological symptoms of preslaughter stress to beef longissimus muscle tenderness. J. Anim. Sci..

[9] Hemsworth P., Rice M., Borg S., Edwards L., Ponnampalam E., Coleman G. (2019). Relationships between handling, behaviour and stress in lambs at abattoirs. Animal.

[10] Merkin G.V., Roth B., Gjerstad C., Dahl-Paulsen E., Nortvedt R. (2010). Effect of pre-slaughter procedures on stress responses and some quality parameters in sea-farmed rainbow trout (Oncorhynchus mykiss). Aquaculture.

[11] Gebresenbet G., Wikner I., Bobobee E.Y.H., Maria G., Villarroel M. (2012). Effect of transport time and handling on physiological responses of cattle. J. Agric. Sci. Technol. A.

[12] Zhang L., Yue H., Zhang H., Xu L., Wu S., Yan H., Gong Y., Qi G. (2009). Transport stress in broilers: I. Blood metabolism, glycolytic potential, and meat quality. Poult. Sci..

[13] Pérez M., Palacio J., Santolaria M., Aceña M., Chacón G., Gascón M., Calvo J., Zaragoza P., Beltran J., Garcıa-Belenguer S. (2002). Effect of transport time on welfare and meat quality in pigs. Meat Sci..

[14] Castagnetti C., Pirrone A., Mariella J., Mari G. (2010). Venous blood lactate evaluation in equine neonatal intensive care. Theriogenology.

[15] Ferasin L., Dodkin S.J., Amodio A., Murray J.K., Papasouliotis K. (2007). Evaluation of a portable lactate analyzer (Lactate Scout) in dogs. Vet. Clin. Pathol..

[16] Kaynar O., Karapinar T., Hayirli A., Baydar E. (2015). Reliability of the Lactate Scout point-of-care instrument for the determination of blood l-lactate concentration in sheep. Vet. Clin. Pathol..

[17] Edwards L., Engle T., Correa J., Paradis M., Grandin T., Anderson D. (2010). The relationship between exsanguination blood lactate concentration and carcass quality in slaughter pigs. Meat Sci..

[18] Burfeind O., Heuwieser W. (2012). Validation of handheld meters to measure blood L-lactate concentration in dairy cows and calves. J. Dairy Sci..

[19] Coghe J., Uystepruyst C., Bureau F., Detilleux J., Art T., Lekeux P. (2000). Validation and prognostic value of plasma lactate measurement in bovine respiratory disease. Vet. J..

[20] Karapinar T., Kaynar O., Hayirli A., Kom M. (2013). Evaluation of 4 point-of-care units for the determination of blood L-lactate concentration in cattle. J. Vet. Intern. Med..

[21] Canada Council of Animal Care (CCAC) (2009). CCAC Guidelines on: Animal USE Protocol Review (2009).

[22] Meléndez D.M., Marti S., Haley D.B., Schwinghamer T.D., Schwartzkopf-Genswein K.S. (2021). Effects of conditioning, source, and rest on indicators of stress in beef cattle transported by road. PLoS ONE.

[23] Thoresen S., Tverdal A., Havre G., Morberg H. (1995). Effects of storage time and freezing temperature on clinical chemical parameters from canine serum and heparinized plasma. Vet. Clin. Pathol..

[24] Cray C., Rodriguez M., Zaias J., Altman N.H. (2009). Effects of storage temperature and time on clinical biochemical parameters from rat serum. J. Am. Assoc. Lab. Anim. Sci..

[25] Acierno M.J., Mitchell M.A. (2007). Evaluation of four point-of-care meters for rapid determination of blood lactate concentrations in dogs. J. Am. Vet. Med. Assoc..

[26] Tennent-Brown B.S., Wilkins P.A., Lindborg S., Russell G., Boston R.C. (2007). Assessment of a point-of-care lactate monitor in emergency admissions of adult horses to a referral hospital. J. Vet. Intern. Med..

